# Supplementary material for: Plasmepsin II–III copy number accounts for bimodal piperaquine resistance among Cambodian Plasmodium falciparum
Source: Nat Commun. 2018 May 2;9:1769. doi: 10.1038/s41467-018-04104-z (PMC5931971; doi:10.1038/s41467-018-04104-z)
Supplement: Supplementary file 1 — Supplementary Information [file 41467_2018_4104_MOESM1_ESM.pdf]

Supplementary Information

Plasmepsin II – III Copy Number Accounts For Bimodal Piperaquine Resistance Among Cambodian *Plasmodium Falciparum*

Bopp et al.

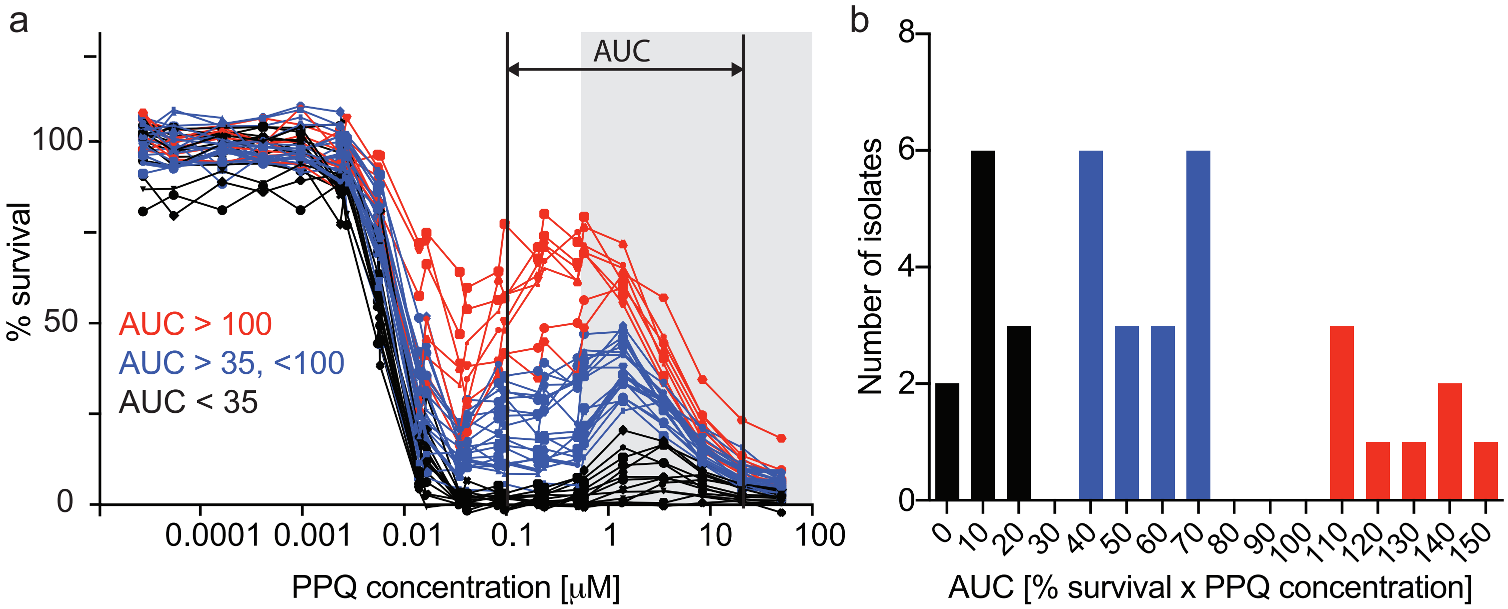

Supplementary Figure 1. Area under the curve (AUC) defines a new PPQ phenotype for 37 culture-adapted isolates. (a) Drug exposure curves for increasing PPQ concentrations are shown. Solid black lines indicate the boundaries of the AUC (0.1 μM to 30 μM), and the shaded gray area indicates data points that were obtained with increased PPQ concentrations compared to standard EC_{50} assays. All drug assays were done in triplicates and repeated two times, shown are the means for each concentration. (b) Frequency distribution of the AUC (in bins of 10) for all 37 isolates tested. These distributions were used to define 3 parasite populations: sensitive (black, AUC <35), intermediate (blue, AUC 35-100), and resistant (red, AUC >100).

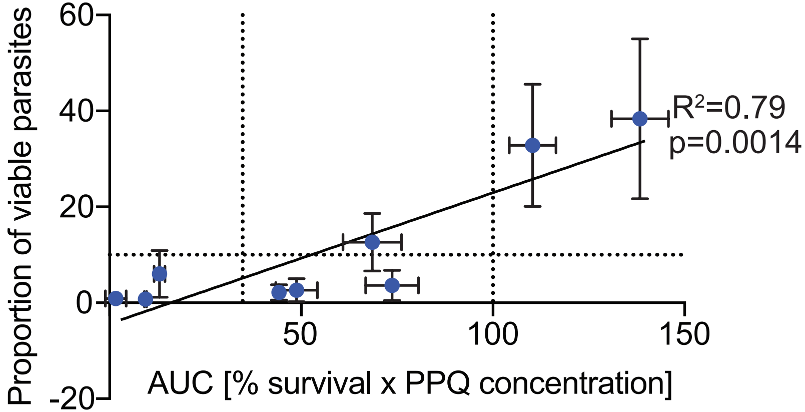

Supplementary Figure 2. AUC correlates with PSA survival rates (Spearman r = 0.85, p = 0.0061). Nine representative isolates (PPQ sensitive, intermediate or resistant) were tested for their PSA survival rates in duplicates (shown are the mean and s.d. N=2 for PSA and N=3 for AUC). Dotted lines are the boundaries of resistance definitions (35 and 100 for AUC, 10% for PSA). The linear regression with R^2 is shown in the graph.

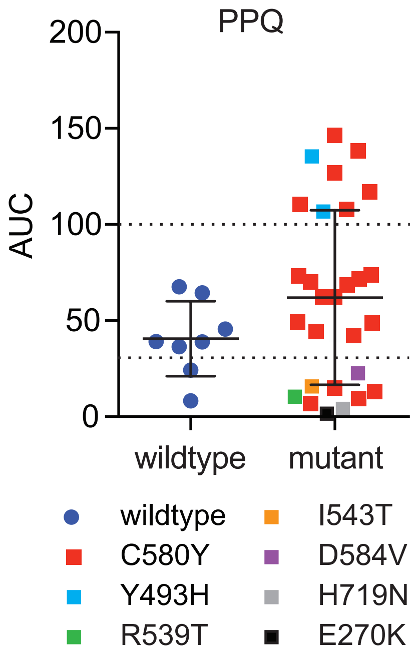

Supplementary Figure 3. PPQ resistance arose on different genetic backgrounds. The AUC values stratified by *Pf*kelch13 mutations are shown (mean +- s.d.). Each dot represents the mean of three biological replicates. Isolates with no *Pf*kelch13 mutations are shown on the left, and isolates with any mutation are shown on the right. The amino acid changes are indicated in the figure. Dotted lines depict the cut off for PPQ resistance according to AUC.

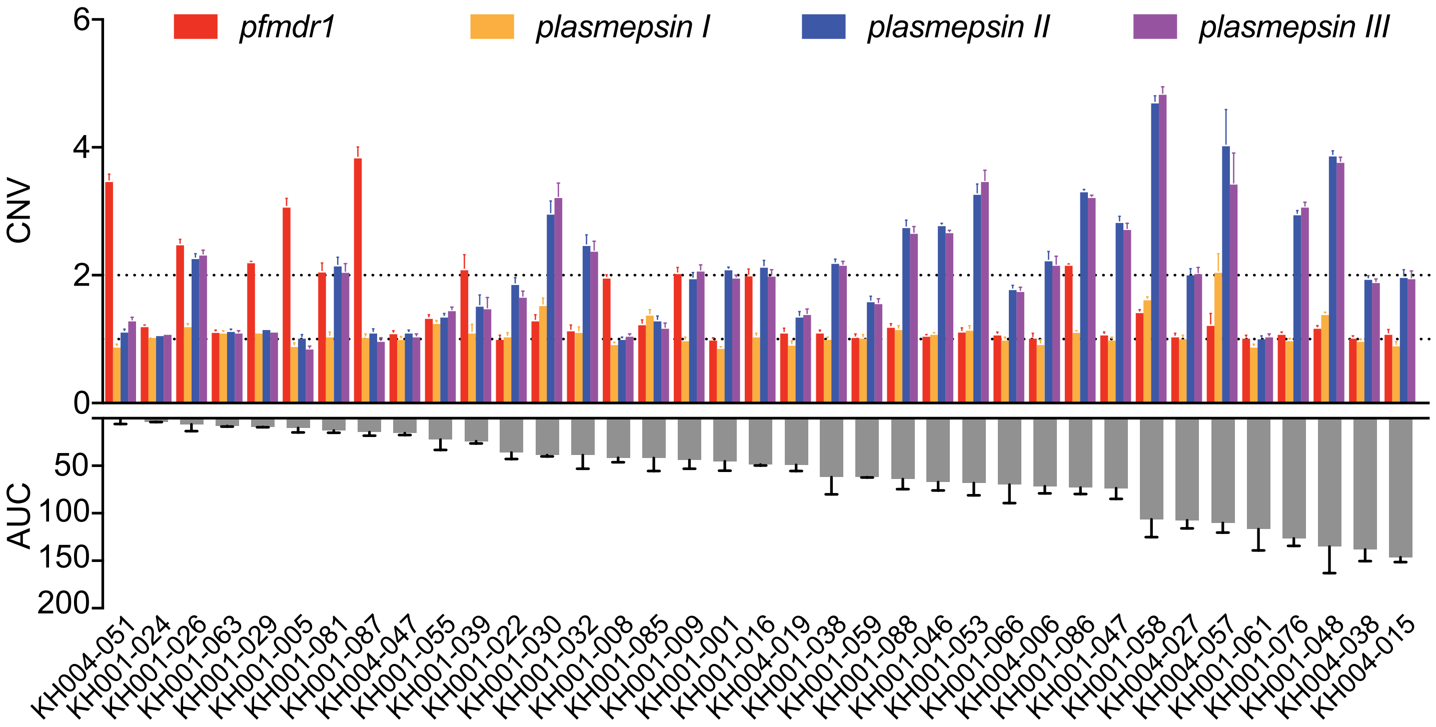

Supplementary Figure 4. Copy number variations (CNVs) of *plasmepsin II – III*, but not *plasmepsin I*, are positively associated with PPQ resistance. For each isolate, the average read depths for *plasmepsin I – III* and *pfmdr1* are shown at the top and the AUC (N=3, mean + s.d.) at the bottom.

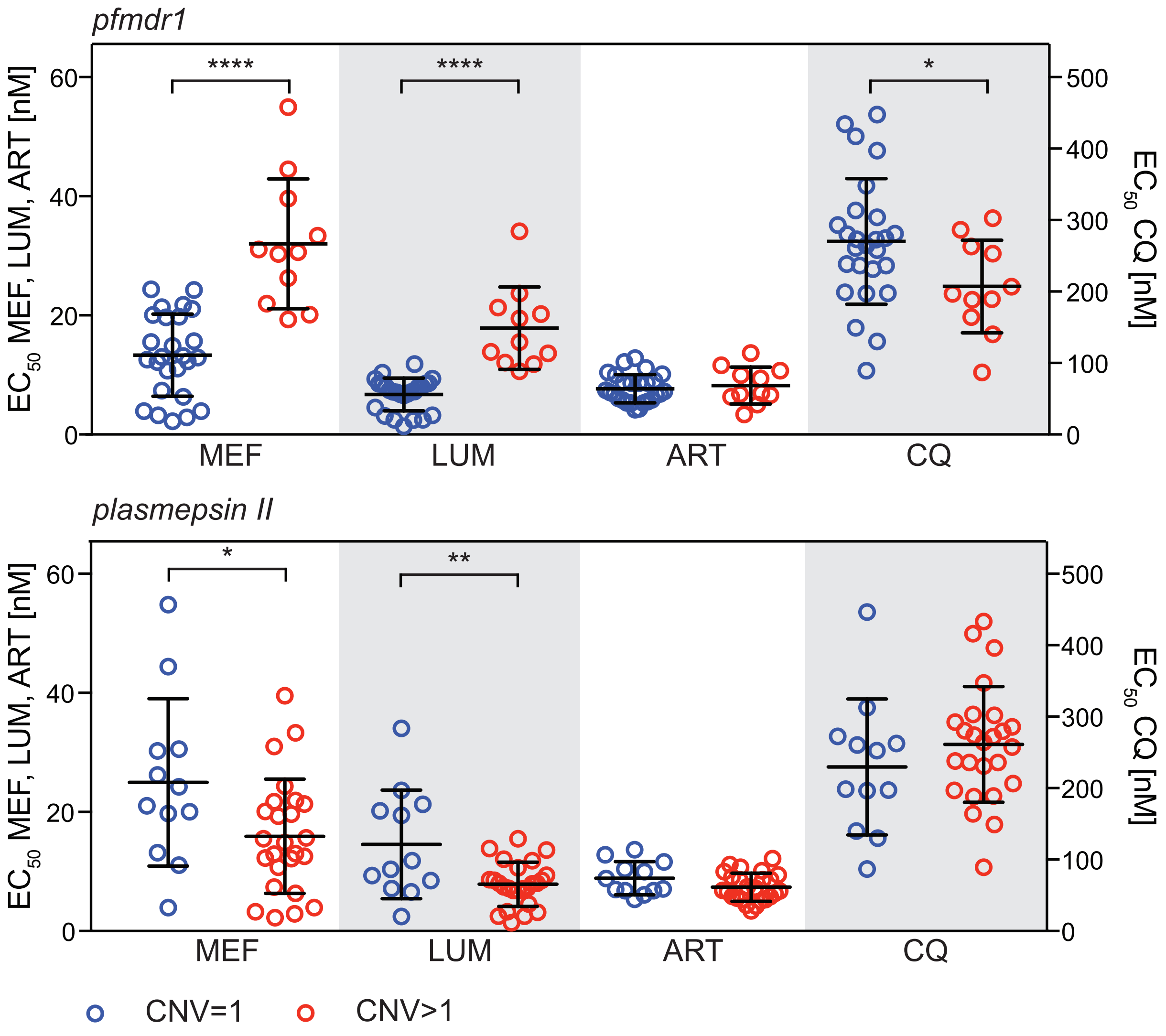

Supplementary Figure 5. Copy number variations (CNVs) for *plasmepsin II* and *pfmdr1* are significantly associated with various drug EC_{50} values. Isolates were grouped according to their *pfmdr1* (top) or *plasmepsin II* (bottom) CNVs with CNVs of 1 in blue and CNVs >1 in red. Mean EC_{50} values from three independent biological replicates run in triplicate for mefloquine (MEF), lumefantrine (LUM), artemisinin (ART) and chloroquine (CQ) are shown. The overall mean and s.d. is shown with black bars, * $p \leq 0.05$, **** $p < 0.0001$, unpaired Student's t test.

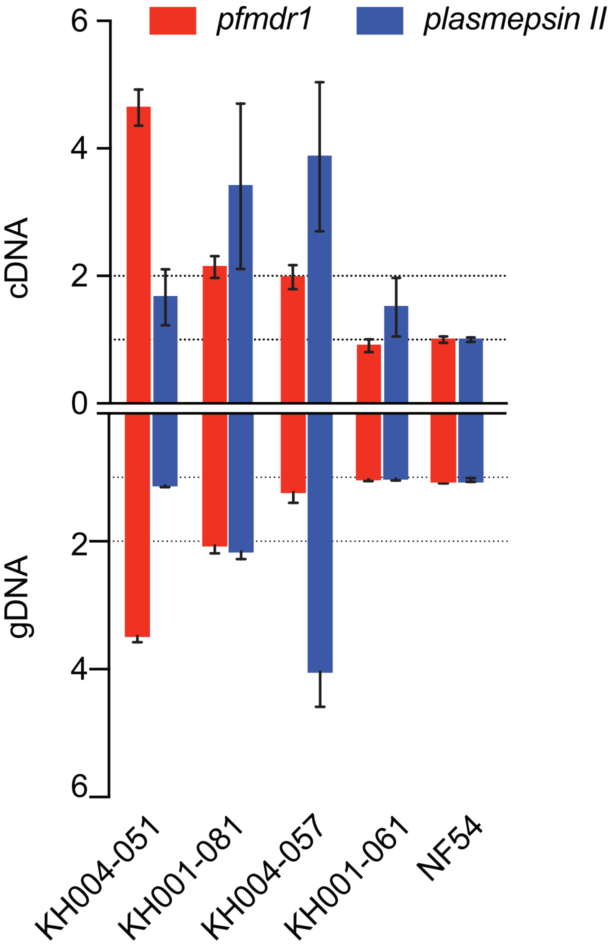

Supplementary Figure 6. *Plasmepsin II* and *pfmdr1* expression tracks with genetic copy numbers. RT-PCR on gDNA (bottom) or cDNA (top) from late-stage parasite isolates was normalized to NF54 (mean and s.d. of three replicates run in quadruplicate). Isolates are ordered according to their AUC (2, 13, 110, and 117, respectively).

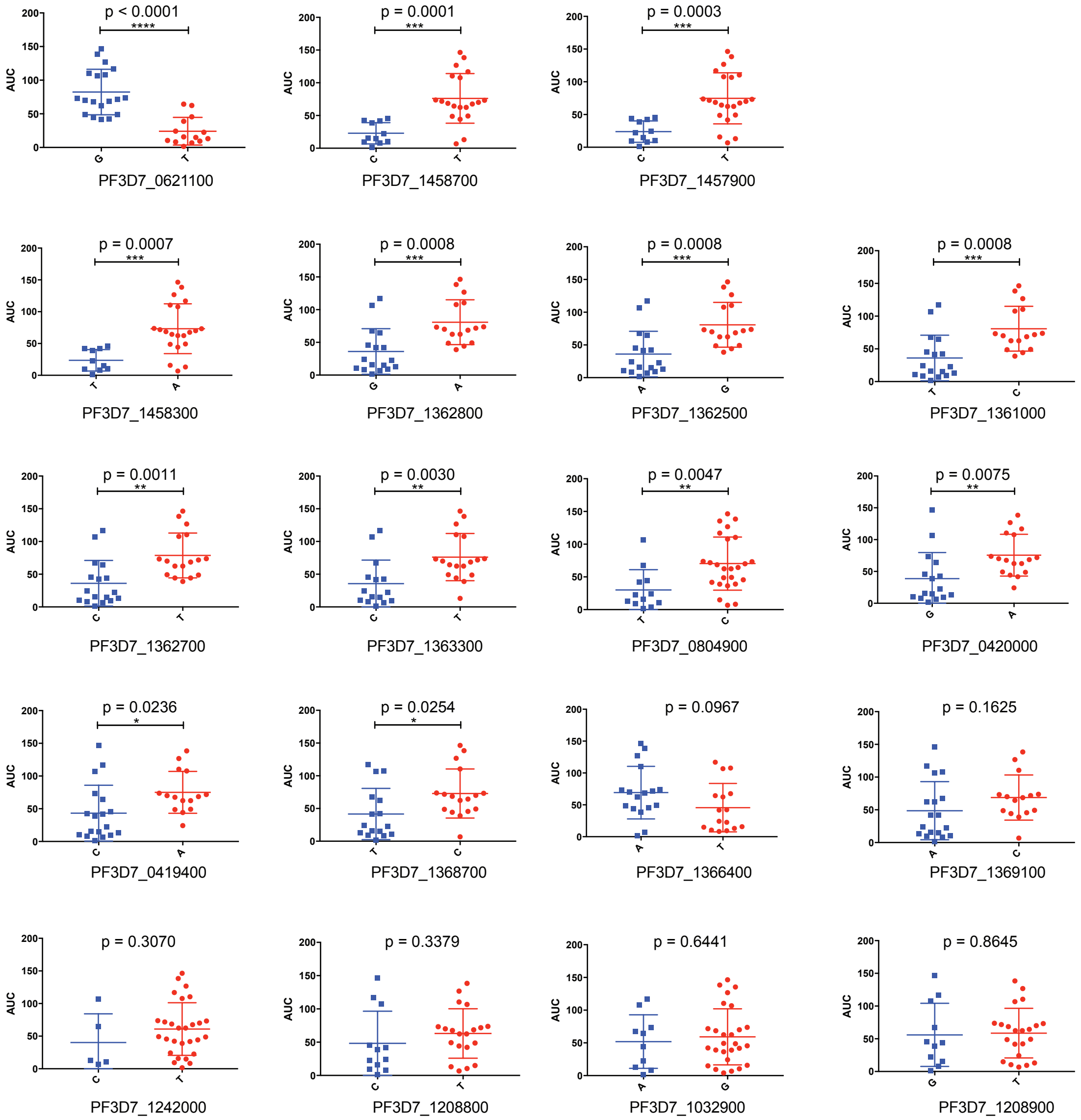

Supplementary Figure 7. AUC for SNPs previously reported to be associated with PPQ resistance. For each SNP position, the AUC was sorted according to the nucleotide position in the isolate. Isolates with more than a third of heterozygous calls were excluded. Shown are the mean and s.d.for the AUC of three biological replicaes run in triplicates. * p≤0.05, ** p<0.01, *** p<0.001, **** p<0.0001, unpaired Student’s t test.

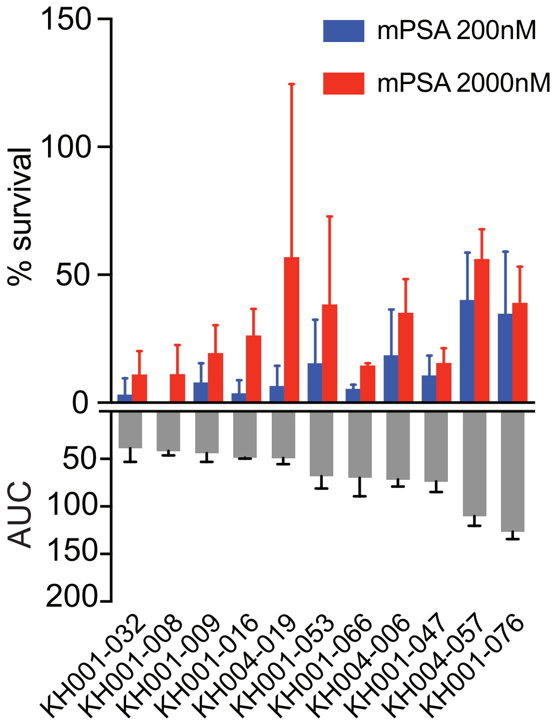

Supplementary Figure 8. Parasites with high AUC survive better under higher PPQ exposure. Parasites with a range of AUC were exposed to either 200 nM (blue) or 2000 nM (red) PPQ or left unexposed, and their survival rates in the modified PSA (mPSA) compared (paired Student's t test, p = 0.0021). Shown is the mean and s.d. of three biological replicates.
