# Supplementary material for: Plasmepsin II–III copy number accounts for bimodal piperaquine resistance among Cambodian Plasmodium falciparum
Source: Nat Commun. 2018 May 2;9:1769. doi: 10.1038/s41467-018-04104-z (PMC5931971; doi:10.1038/s41467-018-04104-z)
Supplement: Supplementary file 3 — Description of Additional Supplementary Files [file 41467_2018_4104_MOESM3_ESM.pdf]

Description of Additional Supplementary Files

File Name: Supplementary Data 1
Description: CNVs for *plasmepsin II – III* and *pfmdr1*.

File Name: Supplementary Data 2
Description: List of all the candidate genes that were used in the associationstudy. These genes all contained SNPs that were previously shown to be strongly associated with PPQR.

File Name: Supplementary Data 3
Description: All SNPs that were reported for *pfcrt*.
